# Raman microspectroscopy: toward a better distinction and profiling of different populations of dental stem cells

**DOI:** 10.3325/CroatMedJ_60_0078

**Published:** 2019-04

**Authors:** Jelena Simonović, Boško Toljić, Božidar Rašković, Vladimir Jovanović, Miloš Lazarević, Maja Milošević, Nadja Nikolić, Radmila Panajotović, Jelena Milašin

**Affiliations:** 1 School of Dental Medicine, University of Belgrade, Belgrade, Serbia; 2 Faculty of Agriculture, Institute of Animal Sciences, University of Belgrade Belgrade, Serbia; 3 Institute for Multidisciplinary Research, University of Belgrade, Belgrade, Serbia; 4 Institute of Physics, University of Belgrade, Belgrade, Serbia

## Abstract

**Aim:**

To characterize stem cells originating from different dental tissues (apical papilla [SCAP], dental follicle [DFSC], and pulp [DPSC]) and test the capacity of Raman microspectroscopy to distinguish between the three dental stem cell types.

**Methods:**

SCAP, DFSC, and DPSC cultures were generated from three immature wisdom teeth originating from three patients. Cell stemness was confirmed by inducing neuro-, osteo-, chondro-, and adipo-differentiaton and by mesenchymal marker expression analysis by flow-cytometry and real-time polymerase chain reaction. Cellular components were then evaluated by Raman microspectroscopy.

**Results:**

We found differences between SCAP, DFSC, and DPSC Raman spectra. The ratio between proteins and nucleic acids (748/770), a parameter for discriminating more differentiated from less differentiated cells, showed significant differences between the three cell types. All cells also displayed a fingerprint region in the 600-700 cm^-1^ range, and characteristic lipid peaks at positions 1440 cm^-1^ and 1650 cm^-1^.

**Conclusion:**

Although different dental stem cells exhibited similar Raman spectra, the method enabled us to make subtle distinction between them.

Dental tissues contain stem cells with high proliferation and differentiation potential, showing great promise for use in regenerative and reparative dentistry, and medicine in general (1,2). Although dental stem cells are multipotent, adult, mesenchymal stem cells (MSCs), originating from the neural crest (3-5), it is difficult to make a precise distinction among the increasing number of newly discovered subpopulations. They rapidly emerge as an attractive biomaterial due to their accessibility and easy isolation compared with embryonic stem cells (ESCs). Dental stem cells (DSCs) can be obtained from several dental tissues, including apical papilla (SCAP), dental pulp of permanent teeth (DPSC), and dental follicle (DFSC) (6).

SCAP can easily be collected after the extraction of immature third molar, from a soft, developing tissue called the apical papilla present at the end of incompletely formed roots. DFSCs are isolated from dental follicle, a sac surrounding the enamel organ and the dental papilla of the developing tooth germ prior to eruption, while DPSC are isolated from the permanent tooth pulp. Although there is a marked resemblance between the three types of cells, they also show some differences in their stemness potential (7-10). An accurate method that would precisely assess stem cell characteristics and help in determining the most appropriate type of cell source for a given regenerative procedure, ie, for the application in different and specific clinical settings, has not yet been established (11).

Raman spectromicroscopy (RS) has been widely used to characterize dental mineral tissues (12-14), showing no apparent negative effects on cells (cellular morphology, proliferation, and other features) due to laser exposure (15-17).

RS has been previously applied to discriminate MSCs from ESCs based on the amount of intracellular lipids (18); to identify various stages of mesenchymal and embryonic murine stem cell differentiation (18-20); and before transplantation, to discriminate normal from abnormal stem cells in *ex vivo* cultures (21). Considering numerous advantages of adult stem cells over ESC, and the growing importance of dental stem cells, we compared DSCs in terms of their structural fingerprint, ie, their biochemical characteristics, by means of Raman spectromicroscopy (RS). The aim of this study was to assess the ability of Raman spectroscopy to discriminate between SCAP, DPSC, and DFSC.

## Material and methods

### Isolation of SCAP, DFSCs and DPSCs

**Figure 1:**
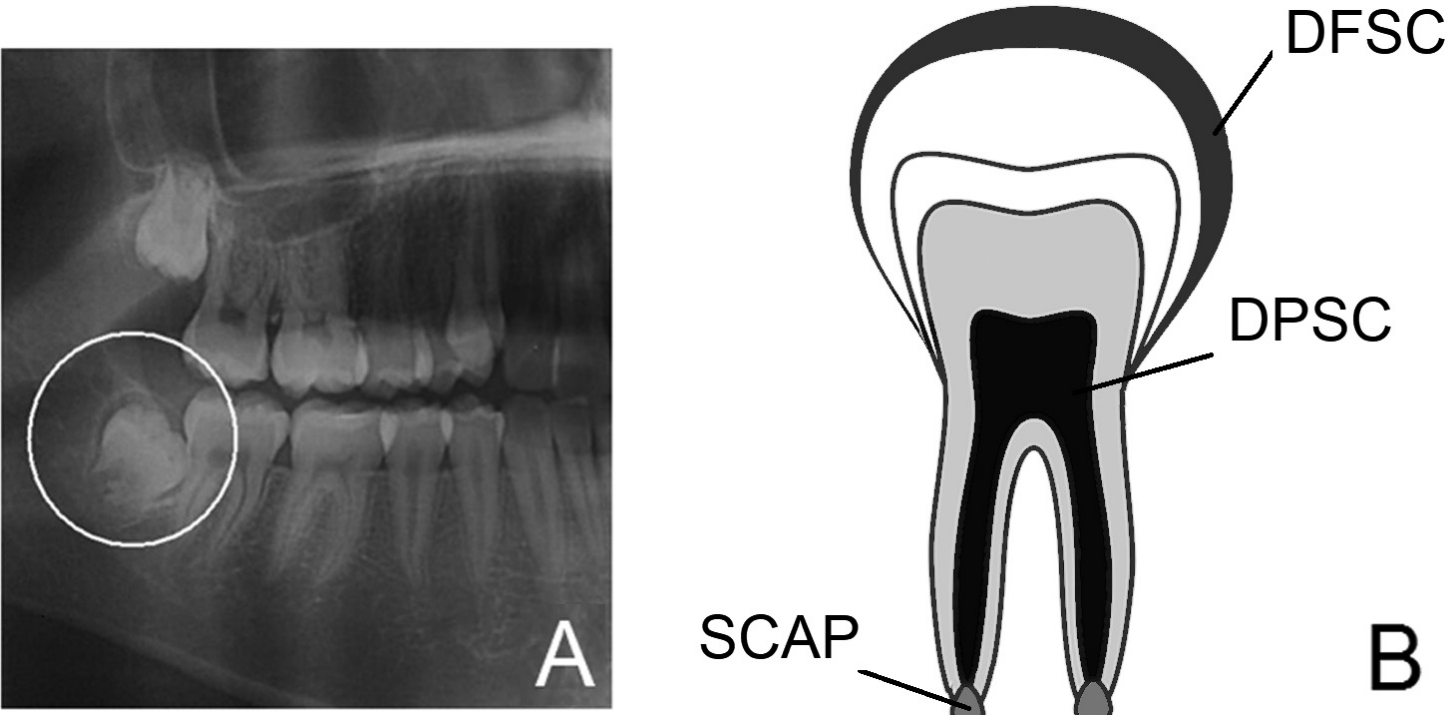
Orthopantomograph of the impacted third molar (**A**) and schematic representation of the three types of tissues used in the analysis: DFSC – dental follicle stem cells; DPSC – dental pulp stem cells; SCAP – apical papila stem cells (**B**); Ctrl – control.

The material was obtained from three immature wisdom teeth (Figure 1), obtained from three patients aged between 14 and 15 years (one tooth per patient). Atraumatical teeth extraction was performed at the Clinic for Oral Surgery, School of Dental Medicine, University of Belgrade, in 2016, after having obtained a written informed consent from the patients’ parents. The study was approved by the Ethics Committee of the School of Dental Medicine, University of Belgrade.

Teeth were immediately transported to the laboratory and further processed under sterile conditions. Tooth surfaces were thoroughly rinsed with Dulbecco’s phosphate buffered saline solution (DPBS, Thermo Fisher Scientific, Waltham, MA, USA), and dental tissues were isolated as previously described (22-24). Briefly, the apical papilla was gently scrapped from the root apex, the dental follicle was separated from the tooth crown with a surgical blade, and the dental pulp tissue was extracted with an endodontic instrument, after having exposed the pulp chamber by crushing the tooth with a sterile clamp. Stem cells were derived without enzymatic digestion (25). Tissues were cut into 1 mm^3^ pieces and separately transferred into Dulbecco’s Modified Eagle Medium (DMEM) supplemented with 10% fetal bovine serum (FBS) and 1% antibiotic-antimycotic solution (all from Thermo Fisher Scientific, Waltham, MA, USA), and cultivated under standard conditions at 37°C and 5% CO_2_. Cell cultures were passaged after reaching 80% confluence. The experiments were done on fifth-passage cells.

### Cell differentiation capacity

To induce neurogenic differentiation, cells were seeded onto T-25 tissue culture flasks in standard culture medium at the density of 1.5 × 10^5^ cells. Control cells were incubated in standard culture medium. After 24 hours, neural pre-induction medium and DMEM with 100 mM beta-mercaptoethanol were added, and cells were incubated for 4 hours. Cell differentiation was continued in a neural induction medium containing recombinant human basic fibroblast growth factor, neural growth factor, and B27 supplement (all from Thermo Fisher Scientific) in DMEM. After 7 days, cell morphology was analyzed under inverted microscope (Primovert Zeiss, Jena, Germany). To induce osteogenic differentiation, cells were seeded in six-well plates with the seeding density of 5 × 10^3^ and cultivated for 28 days in osteogenic differentiation medium (StemPro, Thermo Fisher Scientific). To induce chondrogenic differentiation, cells were seeded in the form of micromass at a total number of 1.6 × 10^6^ and cultivated in a medium for chondrogenic differentiation (StemPro) for 21 days. To induce adipo-differentiation, cells were seeded in six-well plates 1 × 10^4^ cells/cm^2^ and cultivated for 28 days in adipogenic medium (StemPro). To determine successful differentiation, appropriate staining protocols were used. Osteogenic differentiation was confirmed by the presence of mineralization fields stained with 2% Alizarin Red S solution (Centrohem, Belgrade, Serbia); adipogenic differentiation by the presence of neutral lipids stained with 0.5% Oil Red O solution (Sigma Aldrich, Munich, Germany); and chondro-differentiation by the presence of proteoglycan molecules stained with 0.1% Safranin O solution (Centrohem). After staining, the cells were rinsed with DPBS, fixed for 30 minutes with 4% paraformaldehyde, observed under inverted microscope, and photographed.

### Flow cytometry analyses

The markers used for flow-cytometry were fluorescein-isothiocyanate (FITC)-labeled monoclonal antibodies against CD90, CD105, CD34, and phycoerythrin (PE)-labeled mouse monoclonal antibodies against CD73 and CD45. After trypsinization, cells were resuspended in 10% FBS in DPBS (about 1 × 10^6^ cells for every sample). Antibody concentrations were recommended by the manufacturer (Exbio, Prague, Czech Republic). Cells were incubated in the dark for 45 minutes at 4°C with the appropriate combination of antibodies: CD34 (FITC) and CD73 (PE), CD45 (PE) and CD105 (FITC). CD90 (FITC) was added separately. After incubation, cells were rinsed twice with DPBS and analyzed on a multi-laser flow cytometer system (Partec, Munster, Germany).

### Real-time polymerase chain reaction (PCR)

The expression of cell surface mesenchymal markers was assessed by using real-time PCR (qPCR). RNA was isolated by using TRIzol Reagent (Thermo Fisher Scientific), according to manufacturers’ recommendation. Subsequent reverse transcription from 1 μg of total RNA was performed using RevertAid First Strand cDNA Synthesis Kit (Thermo Fisher Scientific) in order to obtain cDNA for qPCR analysis. The list of specific primers (for CD73, CD90, CD45, CD133, and housekeeping gene *GAPDH*) is given in Table 1.

**Table 1:** List of primers used for quantitative polymerase chain reaction

Gene	Sequence of primers (5′→3′)
CD73	Forward: GAGTGGGTGGTCAGAAAATA Reverse: TGCACACTGTTTTTAAGGTG
CD90	Forward: TAACAGTCTTGCAGGTCTCC Reverse: AAGGCGGATAAGTAGAGGAC
CD45	Forward: GCAAAGATGCCCAGTGTTCCACTT Reverse: ATCTGAGGTGTTCGCTGTGATGGT
CD133	Forward: ACTTGGCTCAGACTGGTAAA Reverse: GTTCTGAGCAAAATCCAGAG
*GAPDH*	Forward: TCATGACCACAGTCCATGCCATCA Reverse: CCCTGTTGCTGTAGCCAAATTCGT

The results obtained from each qPCR run were threshold cycle (Ct) values. The relative expression level was assessed using the ΔΔCt method. The relative mRNA expression levels for mesenchymal and hematopoietic markers for each sample were calculated as the ratio between the expression of the gene of interest and the expression of the selected housekeeping gene (*GAPDH*).

### Raman microspectroscopy sample preparation

Cells from the fifth passage were cultivated in growth medium until confluent. After passaging and cell counting, the cells were brought to a concentration of 1 × 10^6^ per mL of the medium. After centrifugation at 300 g for 6 minutes at room temperature, cell pellets were transferred to a golden plate for Raman spectromicroscopy, without fixation.

### Spectroscopic measurements

The Raman spectra of pellets were recorded in the range from 400-2600 cm^-1^ with a Horiba Jobin Yvon Xplora device (Horiba Jobin Yvon S.A.S., Villeneuve-d'Ascq, France) equipped with a BX51 microscope (Olympus, Tokyo, Japan). Raman scattering was excited by a laser diode at the wavelength of 785 nm, with a laser power of 90 mW incident on the pellets and the spot size of around 2 μm. Before spectra acquisition was started, the pellet upper surface was visualized and focused by using a microscope with a lens of 100 × magnification. Each pellet was measured by the Raman device at 25 different spots by using a random movement in order to obtain mean spectra of the sample. The acquisition time per spectrum was 100 s. The dispersive spectrometer had an entrance slit of 100 μm and a focal length of 200 mm, grating of 600 lines/mm, and average spectral resolution of 2.5 cm^-1^. The Raman-scattered light was detected by a thermoelectrically cooled CCD camera (Syncerity, Horiba Scientific, Edison, NJ, USA) operating at 213 K. The spectral acquisition was performed by using LabSpec 6 software (Horiba Scientific, Villeneuve-d'Ascq). For the calibration procedure, the spectra of an aspirin (4-acetylsalicylic acid) were measured daily as a reference control and for subsequent data processing. The achieved signal-to-noise ratio was at least 20.

### Data processing

Each Raman spectrum (250 spectra in total, around 25 spectra per cell type per patient) was first corrected by subtracting its baseline, determined as a 4th order polynomial fitted through several characteristic points (at around 425, 615, 1700, 2100, and 2500 cm^-1^) of the spectrum. Peaks in Raman spectra due to cosmic rays were removed. Then, all spectra were smoothed by Savitzky-Golay filter, using a second-order polynomial. After smoothing, vector normalization was applied to all spectra between 400 and 1800 cm^-1^. Mean and standard deviation was determined for normalized spectra for each cell type (around 75 spectra per cell type): SCAP, DFSC and DPSC.

### Statistical analysis

The normality of distribution was assessed by using Kolmogorov-Smirnov test. The differences between Raman spectra intensities were determined by one way analysis of variance (ANOVA) or Kruskal-Wallis' H-test, followed by Tukey's post-hoc analysis or Bonferroni corrected Mann-Whitney U test, where appropriate. The level of significance was set at *P* = 0.05. For Bonferroni corrected Mann-Whitney U test we used a stricter probability value (less than 0.017). Statistical analysis was performed using the SPSS 17.0 statistical package (SPSS, Chicago, IL, USA).

## Results

### Multilineage differentiation

**Figure 2:**
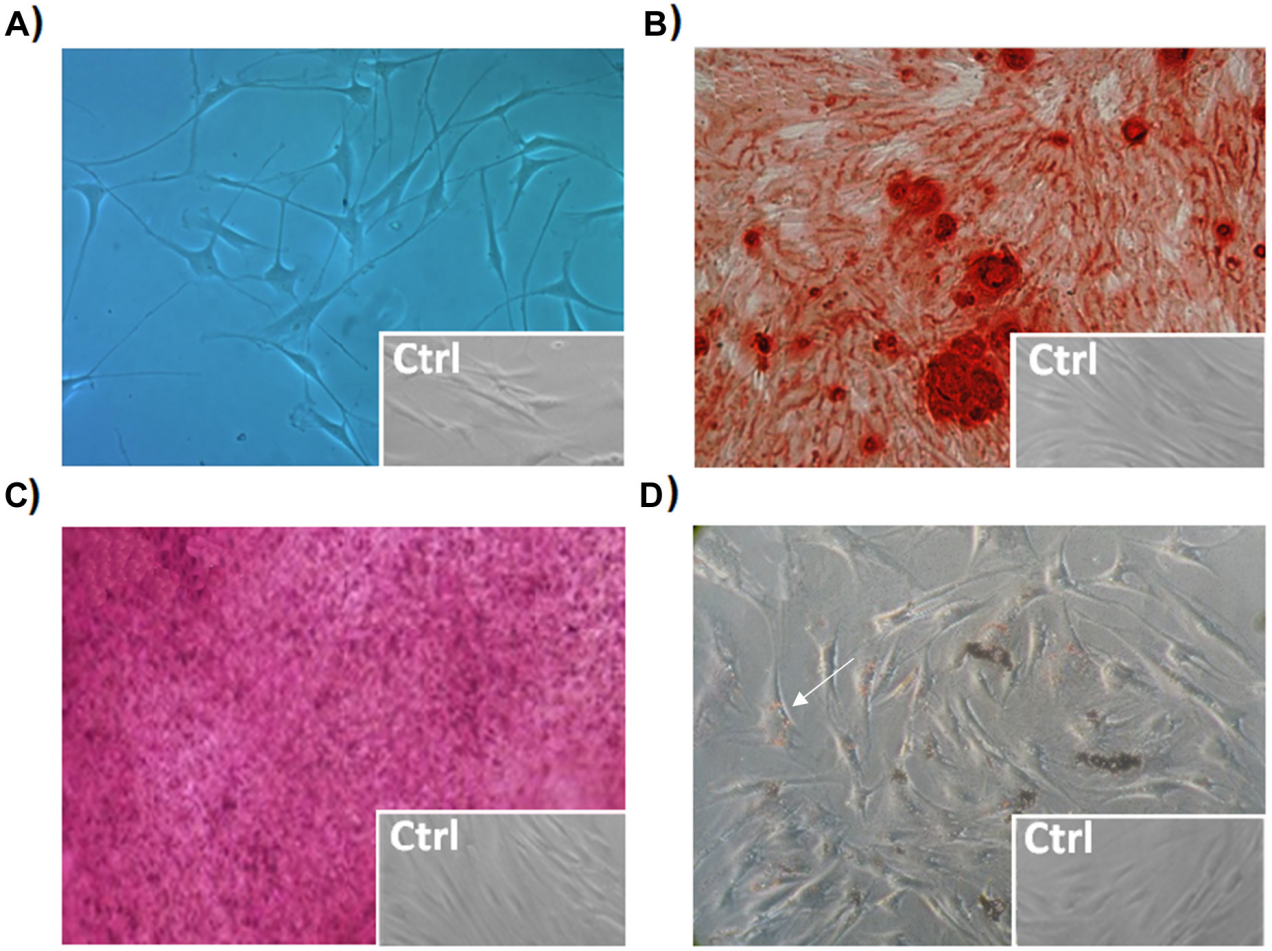
Representative examples of dental stem cell (apical papila) differentiation into four different lineages (magnification 100 × ). Slender projections indicated neuro-differentiation (**A**); Alizarin Red S stained extracellular mineral deposits indicated osteogenic differentiation (**B**); Safranin O stained areas with proteoglycan presence indicated chondrogenic differentiation (**C**); and positive Oil Red O staining indicated lipid droplets accumulation, ie, adipogenic differentiation (**D**).

Specific cell morphology confirmed neurogenic differentiation; Alizarin Red S staining of mineralized nodules around cells confirmed osteogenic differentiation; the presence of Oil Red O staining showed intracellular lipid accumulation; and the presence of Safranin O clusters of proteoglycans characteristic for cartilage cells confirmed chondro-differentiation (Figure 2). Cells of all the three origins displayed comparable behavior when induced toward a specific lineage. In the control group (non-induced cells) there were no stained cells.

### Cell surface markers detection by flow-cytometry

**Figure 3:**
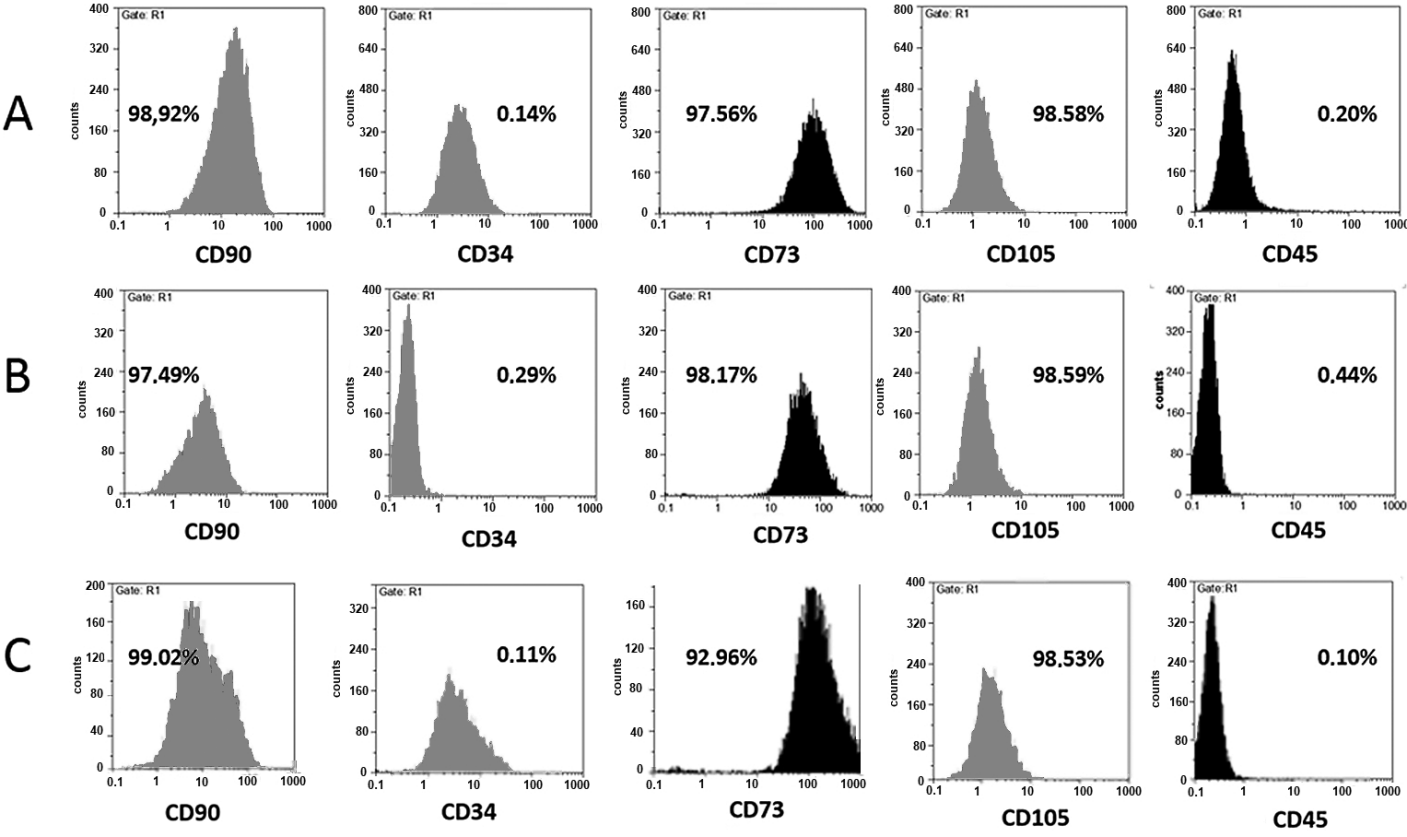
Immunophenotypic profile of mesenchymal stem cells derived from (**A**) dental pulp, (**B**) dental follicle, and (**C**) apical papilla, all strongly positive for CD90, CD73, and CD105 (markers associated with mesenchymal stem cells) and negative for CD45 and CD34 (markers of hematopoietic cells).

SCAP, DFSC, and DPSCs were strongly positive for CD90, CD73, and CD105 (cell surface markers of mesenchymal stem cells) and negative for CD45 and CD34 (cell surface markers of hematopoietic cells) (Figure 3). No significant difference was observed between different cell types based on flow-cytometry.

### Gene expression analysis by real-time PCR

**Figure 4:**
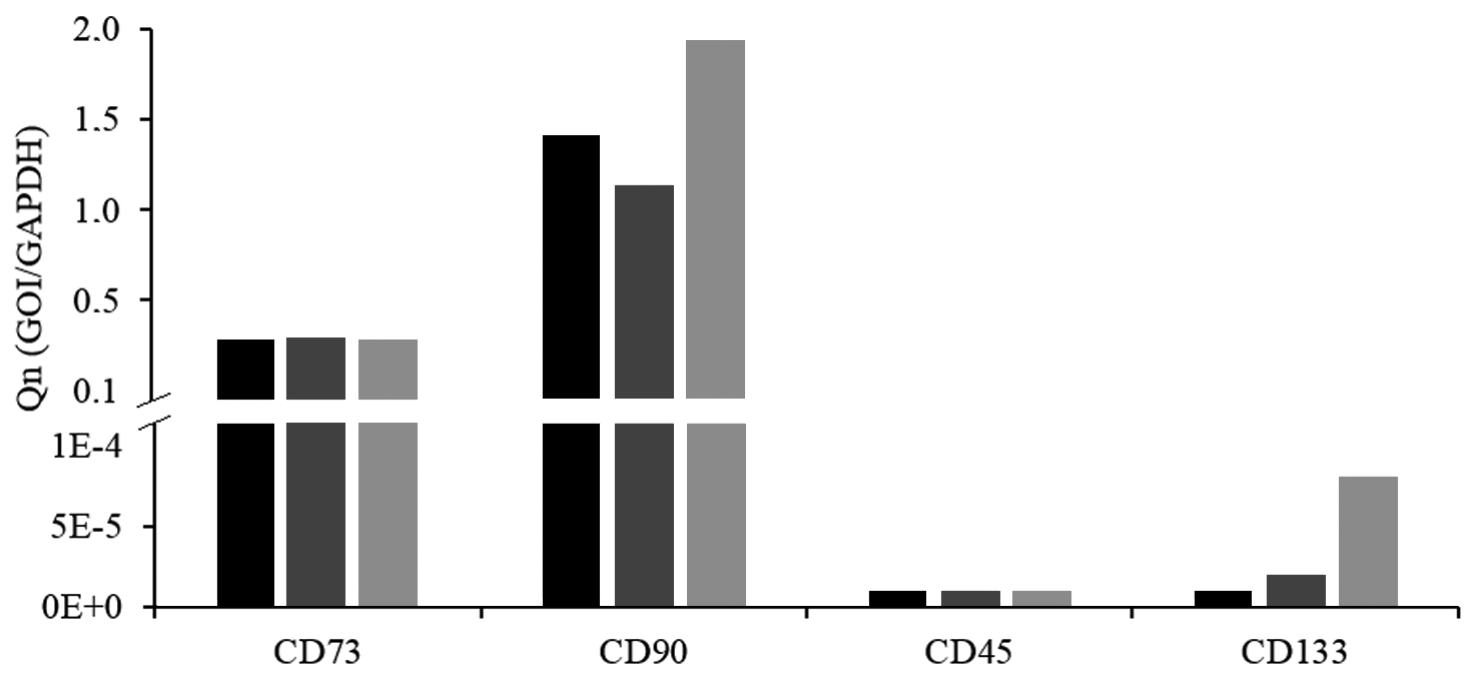
Relative gene expression of mesenchymal (CD73 and CD90) and hematopoietic markers (CD45 and CD133) of stem cells isolated from dental pulp (DPSC, black), apical papilla (SCAP, dark gray), and dental follicle (DFSC, light gray).

The main mesenchymal markers expression was confirmed in all three cell groups, without significant difference between the cells, while the expression of hematopoetic markers was negligible in all samples (Figure 4).

### Raman spectromicroscopy

**Figure 5:**
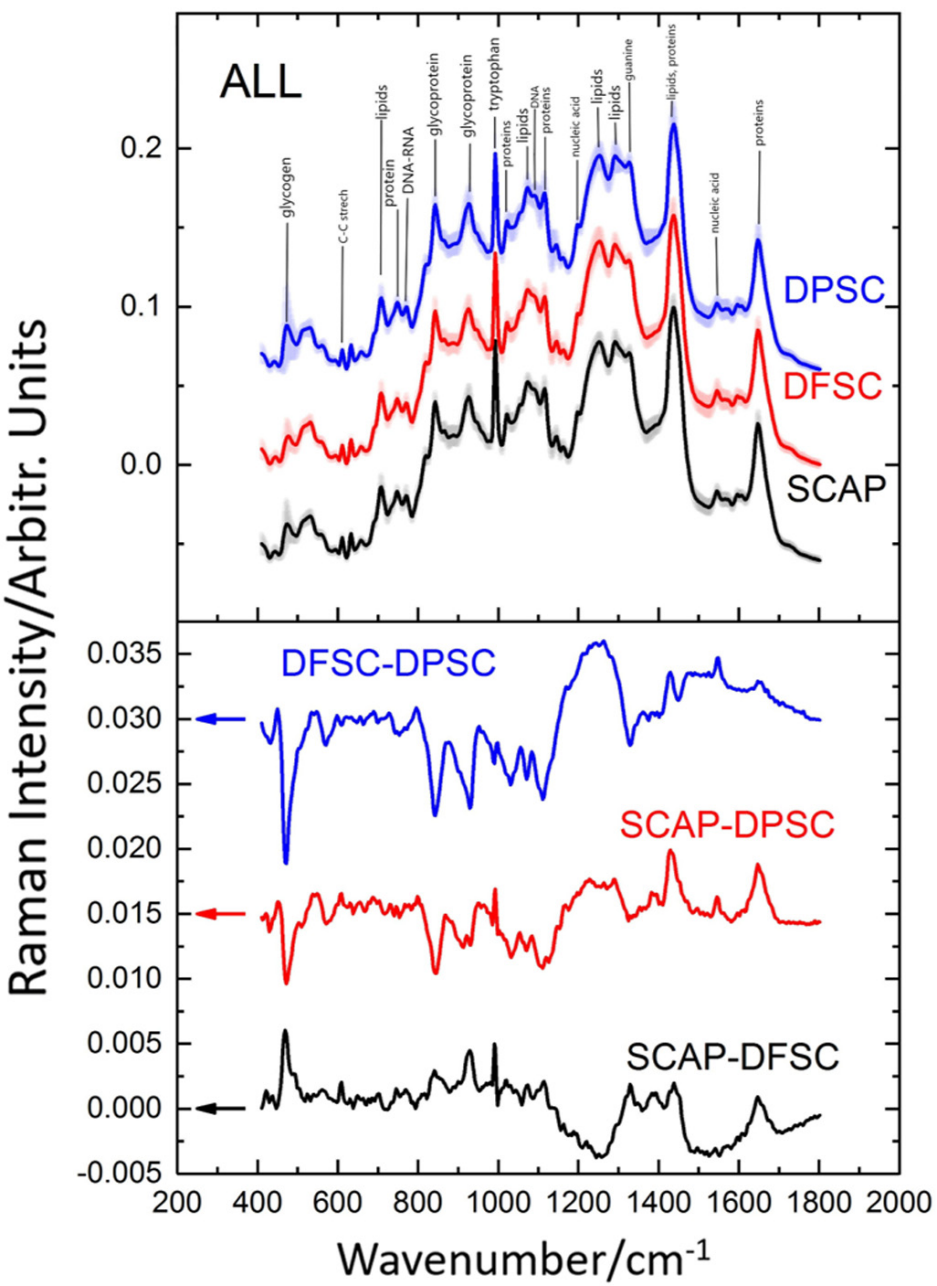
Raman spectra of different cell types averaged over all three subjects (75 spectra per cell type), offset for clarity. Shaded regions mark the standard deviation of spectra (upper panel); spectra subtracted from each other to emphasize the differences (lower panel).

Cell spectra of the three patients, when averaged, showed obvious similarities (Figure 5). However, there were significant differences between SCAP, DFSC, and DPSC (Table 2). Generally speaking, the most important differences were noticed between DFSC and DPSC; namely, out of 20 prominent peaks, 11 showed significant differences. Significant differences between SCAP and DFSC were observed in 8 peaks, while significant differences between SCAP and DPSC were observed only in 4 peaks. The parameter R4 (the ratio between proteins and nucleic acids, 748/770), which is considered to be a reliable parameter for the discrimination between more and less differentiated cells (15), showed a significant difference between the three cell types (SCAP vs DFSC, *P* = 0.018; SCAP vs DPSC, *P* < 0.001; DFSC vs DPSC, *P* = 0.009). From the R4 values it can tentatively be concluded that the decreasing potency of the analyzed cells was: SCAP>DFSC>DPSC.

**Table 2:** Biochemical differences between apical papilla (SCAP), dental follicle (DFSC), and pulp stem cells (DPSC) determined by Raman spectroscopy and vector-normalized

Wavenumber (cm^-1^)/ assignment	Raman intensity peaks in				*P*-values			
	SCAP		DFSC	DPSC	SCAP vs DFSC	SCAP vs DPSC		DFSC vs DPSC
471 glycogen*	-0.043 (0.021)	-0.047 (0.012)		-0.047 (0.038)	0.008	0.501	0.166	
612 C-C stretch	-0.046 ± 0.002	-0.048 ± 0.002		-0.047 ± 0.002	<0.001	0.001	0.376	
706 lipids*	-0.013 (0.011)	-0.013 (0.012)		-0.014 (0.013)	NS	NS	NS	
748 protein	-0.017 ± 0.003	-0.018 ± 0.004		-0.017 ± 0.004	NS	NS	NS	
770 DNA-RNA	-0.026 ± 0.004	-0.018 ± 0.005		-0.017 ± 0.003	<0.001	<0.001	0.950	
843 glycoprotein	0.040 ± 0.006	0.037 ± 0.009		0.044 ± 0.010	0.079	0.002	<0.001	
929 glycoprotein	0.042 ± 0.008	0.038 ± 0.008		0.044 ± 0.012	0.010	0.267	<0.001	
990 tryptophan	0.078 ± 0.007	0.074 ± 0.008		0.077 ± 0.011	0.004	0.392	0.131	
1023 proteins	0.032 ± 0.005	0.030 ± 0.006		0.034 ± 0.007	0.111	0.024	<0.001	
1074 lipids	0.053 ± 0.005	0.051 ± 0.006		0.055 ± 0.009	0.194	0.041	<0.001	
1094 DNA*	0.045 (0.020)	0.045 (0.006)		0.046 (0.012)	0.108	0.164	0.006	
1116 protein*	0.046 (0.010)	0.043 (0.010)		0.045 (0.020)	0.016	0.495	0.002	
1198 nucleic acid*	0.033 (0.010)	0.035 (0.010)		0.031 (0.000)	0.028	0.004	<0.001	
1252 lipids	0.078 ± 0.006	0.081 ± 0.008		0.075 ± 0.008	0.010	0.163	<0.001	
1293 lipids*	0.080 (0.010)	0.081 (0.010)		0.079 (0.010)	0.086	0.194	0.004	
1329 guanine	0.070 ± 0.004	0.068 ± 0.004		0.070 ± 0.006	0.043	0.978	0.023	
1440 lipids, proteins*	0.100 (0.010)	0.096 (0.020)		0.100 (0.020)	NS	NS	NS	
1546 nucleic acid	0.091 ± 0.004	0.090 ± 0.006		0.089 ± 0.008	<0.001	0.269	<0.001	
1650 proteins	0.026 ± 0.008	0.025 ± 0.008		0.022 ± 0.010	0.816	0.020	0.088	
748/770	0.809 ± 0.168	0.888 ± 0.204		0.972 ± 0.176	0.018	<0.001	0.009	

*Data are presented as mean ± standard deviation or median (inter-quartile range). *P* values represent differences between groups determined by one-way analysis of variance (ANOVA) or Kruskal-Wallis' H-test*, followed by Tukey's post-hoc analysis or Mann-Whitney U test, where appropriate. Bonfferoni’s correction was applied after Kruskal-Wallis' H-test.

## Discussion

In the present study, the use of standard methodologies for quantitative and qualitative estimation of stemness characteristics of dental tissue cells suggested that SCAP, DFSC, and DPSC exhibited very similar phenotypic characteristics during cultivation and differentiation. Although different dental stem cells exhibited similar Raman spectra, the method enabled us to make a subtle distinction between them.

Several cellular components are closely related to stemness characteristics. Stem cells must constantly maintain a fine balance between anabolism and catabolism, and metabolic plasticity is seen as a crucial phenomenon in the regulation of stem cell transition from self-renewal to lineage specification (26). For instance, glycogen is considered a regulator of potency and cellular differentiation capacity. Although the function of increased storage and production of glycogen in human stem cells is not fully understood, glycogen synthesis seems to be crucial for self-renewal, cell survival, growth rates, shorter doubling time, and differentiation (27,28). High glycogen accumulation is also typically observed in human embryonic stem cells, though its level has not been fully investigated in other stem cells types (29). These findings are in line with the present study, as Raman peaks for glycogen/glycoproteins at 470, 841, and 927 cm^-1^ showed substantial intensities in all cell types.

Lipids are also considered to be closely linked to stem cells potency (20,30), and lipid metabolism has a pivotal role in stem cell fate determination (31,32). Namely, inhibition of the eicosanoid pathway is associated with the maintenance of the pluripotent state in murine ESC (32). The eicosanoid pathway promotes the hydrolysis of membrane phospholipids by releasing lipid messengers into the cytoplasm (32), and their level progressively decreases during differentiation (18). In our study, all three cell types in all patients exhibited substantial lipid levels as judged by the very characteristic peaks at 1440 and 1650 cm^-1^.

Nucleic acids content could also be considered as a marker of stemness (33,34), as well as the ratio between protein (tryptophan) and nucleic acids. While proteins have more prominent peaks in differentiated cells, nucleic acids quantity, on the contrary, decreases during differentiation, ie, there is a dominance-reversal in differentiated cells. Tryptophan (protein) peak vs nucleic acid peak (748 vs 770 cm^-1^) ratio is therefore considered as a differentiation status indicator (18,35). In the present study, highly significant differences in this ratio were obtained between the three cell types. As judged by R4, the decreasing differentiation potential of the three types of cells was as follows: SCAP>DFSC>DPSC. This result, however, must be interpreted with caution, since it can probably vary depending on the patient's age and the stage of tooth development. Further studies, on a larger sample and on other cell populations would also be necessary for final conclusions to be drawn.

New cell subpopulations are emerging, especially in the orofacial region (36), necessitating the use of different techniques that are able to distinguish among them in order to better understand their lineage relationships. Raman microspectroscopy can provide a rapid, non-invasive, and label-free tool for uncovering subtle biochemical differences that can be used to distinguish more potent from less potent stem cells. The present study brings new insights into dental stem cell characteristics, enhancing the possibility of their clinical application.
